# Inhibition of Phosphodiesterase 3A by Cilostazol Dampens Proinflammatory Platelet Functions

**DOI:** 10.3390/cells10081998

**Published:** 2021-08-05

**Authors:** Daniëlle M. Coenen, Alexandra C. A. Heinzmann, Silvia Oggero, Hugo J. Albers, Magdolna Nagy, Perrine Hagué, Marijke J. E. Kuijpers, Jean-Marie Vanderwinden, Andries D. van der Meer, Mauro Perretti, Rory R. Koenen, Judith M. E. M. Cosemans

**Affiliations:** 1 Department of Biochemistry, Cardiovascular Research Institute Maastricht (CARIM), Maastricht University, 6229 ER Maastricht, The Netherlands; danielle.coenen@uky.edu (D.M.C.); a.heinzmann@maastrichtuniversity.nl (A.C.A.H.); m.nagy@maastrichtuniversity.nl (M.N.); marijke.kuijpers@maastrichtuniversity.nl (M.J.E.K.); r.koenen@maastrichtuniversity.nl (R.R.K.); 2 Department of Molecular and Cellular Biochemistry, University of Kentucky College of Medicine, Lexington, KY 40506, USA; 3 Biochemical Pharmacology, William Harvey Research Institute, Queen Mary University of London, London E1 4NS, UK; s.oggero@qmul.ac.uk (S.O.); m.perretti@qmul.ac.uk (M.P.); 4 BIOS Lab-on-a-Chip Group, Technical Medical Centre, MESA+ Institute for Nanotechnology, University of Twente, 7522 NB Enschede, The Netherlands; h.j.albers@utwente.nl; 5 Applied Stem Cell Technologies Group, Technical Medical Centre, University of Twente, 7522 NB Enschede, The Netherlands; andries.vandermeer@utwente.nl; 6 Laboratory of Neurophysiology, Faculty of Medicine, Université Libre de Bruxelles, B-1070 Brussels, Belgium; perrine.hague@ulb.ac.be (P.H.); jmvdwin@ulb.ac.be (J.-M.V.)

**Keywords:** platelets, thrombosis, vascular inflammation, phosphodiesterase inhibitors, extracellular vesicles

## Abstract

Objective: platelets possess not only haemostatic but also inflammatory properties, which combined are thought to play a detrimental role in thromboinflammatory diseases such as acute coronary syndromes and stroke. Phosphodiesterase (PDE) 3 and -5 inhibitors have demonstrated efficacy in secondary prevention of arterial thrombosis, partially mediated by their antiplatelet action. Yet it is unclear whether such inhibitors also affect platelets’ inflammatory functions. Here, we aimed to examine the effect of the PDE3A inhibitor cilostazol and the PDE5 inhibitor tadalafil on platelet function in various aspects of thromboinflammation. Approach and results: cilostazol, but not tadalafil, delayed ex vivo platelet-dependent fibrin formation under whole blood flow over type I collagen at 1000 s^−1^. Similar results were obtained with blood from *Pde3a* deficient mice, indicating that cilostazol effects are mediated via PDE3A. Interestingly, cilostazol specifically reduced the release of phosphatidylserine-positive extracellular vesicles (EVs) from human platelets while not affecting total EV release. Both cilostazol and tadalafil reduced the interaction of human platelets with inflamed endothelium under arterial flow and the release of the chemokines CCL5 and CXCL4 from platelets. Moreover, cilostazol, but not tadalafil, reduced monocyte recruitment and platelet-monocyte interaction in vitro. Conclusions: this study demonstrated yet unrecognised roles for platelet PDE3A and platelet PDE5 in platelet procoagulant and proinflammatory responses.

## 1. Introduction

In atherosclerosis and its major clinical presentation, myocardial infarction and stroke, there is a strong crosstalk between inflammatory and thrombotic processes. For instance, platelets are considered to promote atherogenesis by recruiting leukocytes to the inflamed endothelium (via chemokines) and by triggering an inflammatory response in endothelial cells through direct interaction or with released extracellular vesicles (EVs) [1]. Prevention of recurrent cardiovascular events predominantly comprises therapy with platelet activation inhibitors, such as aspirin and clopidogrel, or dual pathway inhibition with a low dose anticoagulant on top of aspirin [2]. Despite this comprehensive treatment, a quarter of the yearly arising strokes and myocardial infarcts are reoccurring [3,4], highlighting a need for new or additional treatment options.

In contrast to standard antiplatelet and anticoagulant drugs, which dampen platelet or coagulant activity, phosphodiesterase (PDE) inhibitors act by promoting vasodilation and platelet inhibitory pathways [5]. The PDEs 2, 3, and 5 are expressed in platelets and hydrolyse cAMP (PDE2, PDE3) and cGMP (PDE2, PDE3, PDE5) to adenosine monophosphate and guanosine monophosphate, respectively, thereby lowering the threshold for platelet activation [5]. Whereas PDE2 inhibitors are still under preclinical development, the PDE3A inhibitor cilostazol (IC_50_ PDE3 inhibition: 0.2 μM) is prescribed to alleviate symptoms of intermittent claudication in patients with peripheral artery disease and for secondary stroke prevention [6,7,8]. Notably, cilostazol, as opposed to aspirin and clopidogrel, does not influence bleeding time [9,10]. The PDE5 inhibitor dipyridamole was formerly used as a standard treatment for secondary stroke treatment in combination with aspirin, but current guidelines now advise monotherapy with aspirin or with clopidogrel [11,12,13,14]. Dipyridamole (IC_50_ PDE5 inhibition: 0.9 μM) is known for its antithrombotic action. However, the platelet inhibiting function of dipyridamole does not only rely on its action via PDE5, but also on the blockage of adenosine reuptake [15]. In contrast to dipyridamole, tadalafil (IC_50_ PDE5 inhibition: 1.8 nM), sildenafil (IC_50_ PDE5 inhibition: 5.22 nM), and vardenafil (IC_50_ PDE5 inhibition: 0.7 nM) have a high specificity in only inhibiting PDE5, and these compounds are used for the treatment of erectile dysfunction [5]. Tadalafil and sildenafil are also beneficial in pulmonary arterial hypertension [16]. The effect of PDE3 inhibition on haemostatic or thrombotic platelet activation and aggregation has been explored widely, but research on cilostazol in platelet proinflammatory function is limited. Moreover, research about PDE5 inhibition with its specific inhibitor tadalafil with regard to a thrombotic or inflammatory state is scarce.

Here, we examine the effect of the PDE3A inhibitor cilostazol and the PDE5 inhibitor tadalafil on platelet function in various aspects of thromboinflammation.

## 2. Methods

### 2.1. Blood Collection

Human blood was obtained from healthy donors after full informed consent in compliance with the Declaration of Helsinki. Studies were approved by the local Medical Ethics Committee. Users of antiplatelet and/or anticoagulant medication were excluded. Regarding the flow experiments under inflammatory conditions, human whole blood was kindly provided by the Experimental Centre for Technical Medicine at the University of Twente.

### 2.2. Mice

Animal experiments were approved by the Ethics Committee for Animal Well-Being of the Faculty of Medicine, Université Libre de Bruxelles (ULB), protocol LA1230331-621N, in line with the regional and national regulations and the EU directives. Mice were bred and maintained under standard husbandry conditions and a regular diet in the animal facility of the Faculty of Medicine, ULB. Genotyping was performed as described [17]. Male and female C57BL/6 wild type (WT) and *Pde3a* deficient (knockout; KO) mice between 10 and 26 weeks old were anaesthetized by intraperitoneal injection with 800 μL avertin, which was the standard procedure approved by the Ethical Committee for the procurement of biological materials for ex vivo studies. The effects of the anaesthesia were verified by checking the foot reflex. No postanaesthetic effects were present as the mice were directly euthanized by cervical dislocation after blood collection on 3.2% sodium citrate via retro-orbital puncture.

### 2.3. Cell Culture

Human umbilical vein endothelial cells (HUVECs) were cultured in ECGM medium (PromoCell) in a humidified atmosphere with 5% CO_2_ at 37 °C. HUVECs between passage 5 and 7 were used.

Human acute monocytic leukaemia (THP-1) cells were cultured in RPMI-1640 GlutaMAX medium supplemented with 20% FCS and 1% penicillin/streptomycin in a humidified atmosphere with 5% CO_2_ at 37 °C. THP-1 cells between passage 5 and 16 were used.

### 2.4. Statistical Analysis

Flow perfusion experiments over collagen were analysed with a two-way ANOVA, except for the time to fibrin formation with the pharmacological intervention, which was tested with an ordinary one-way ANOVA. Paired *t*-tests were performed for flow perfusion experiments over inflamed endothelium. The experiments regarding THP-1 migration, cAMP/cGMP levels and VASP phosphorylation were analysed with a Kruskal–Wallis test. THP-1 adhesion was evaluated with a Wilcoxon matched-pairs signed rank test. Statistical testing for the remaining experiments was performed using an ordinary one-way ANOVA. Correction for multiple comparisons was achieved with Dunnett’s, Dunn’s, Sidak’s and Holm-Sidak’s post hoc testing. All data were statistically analysed with Graphpad Prism 8.4.3.

## 3. Results

### 3.1. Function of PDE3A and PDE5 in P-Selectin Expression and α_IIb_β_3_ Activation of Human and Mouse Platelets

**Figure 1:**
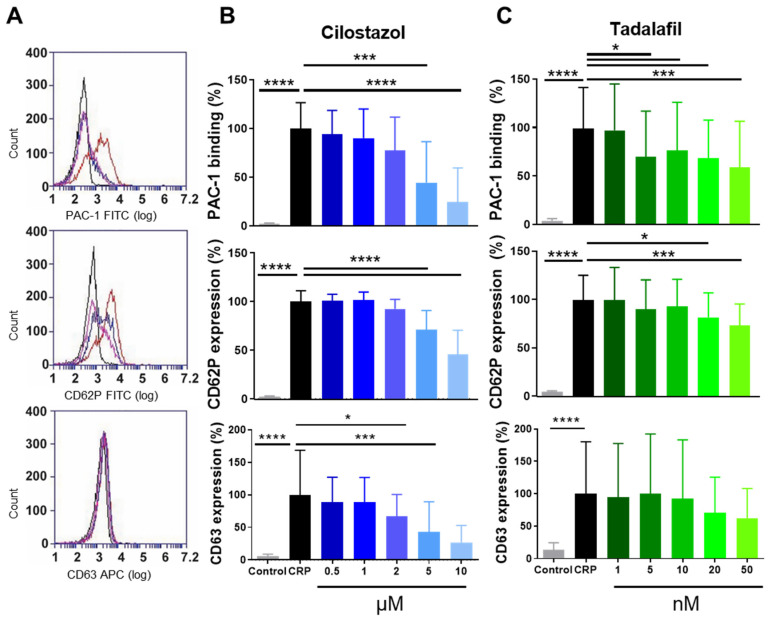
PDE3A and PDE5 inhibition dose-dependently decreases platelet integrin activation and granule secretion. Washed platelets were activated with collagen-related peptide (CRP, 0.3 µg/mL) and platelet integrin α_IIb_β_3_ activation (PAC-1: 65.68 ± 17.42%) and platelet α-granule secretion (CD62P: 72.74 ± 8.17%), dense-granule secretion (CD63: 20.36 ± 13.97%) were measured by flow cytometry (**A**). Dose-dependent decrease by PDE3A (**B**) and PDE5 (**C**) inhibition. Data in duplicate in the percentage normalised against the activated platelets without inhibitor are shown. Corresponding vehicle controls were included for every condition. Histograms: black represents control, red represents CRP, pink represents cilostazol (5 µM), and blue represents tadalafil (10 nM). Mean + S.D., *n* = 5–7, * *p* < 0.05, *** *p* < 0.001, **** *p* < 0.0001. Statistics: ordinary one-way ANOVA followed by Holm-Sidak’s multiple comparisons test. CRP, collagen-related peptide.

To determine the effects of cilostazol and tadalafil on platelet activation markers, washed human platelets were incubated with increasing concentrations of these compounds prior to stimulation with the collagen-analogue CRP (0.3 μg/mL) and analysed by flow cytometry (Figure 1A). In suspension, cilostazol dose dependently inhibited platelet integrin α_IIb_β_3_ activation and the secretion of platelet α- and dense granules (Figure 1B). Tadalafil significantly inhibited platelet integrin activation at 5 nM and above, while secretion of α-granules was only decreased at 20 and 50 nM. Dense granule secretion was not affected at these concentrations (Figure 1C). In resting or CRP-stimulated washed platelets, incubation with cilostazol or with tadalafil neither led to significantly altered cAMP and cGMP levels nor to altered phosphorylation of VASP at serine-157 or -239 (Supplementary Table S1 and Supplementary Figure S2).

Surprisingly, in platelets from WT or *Pde3a* KO mice measured with flow cytometry, integrin α_IIb_β_3_ activation and α-granule secretion were unaltered upon stimulation of diluted whole blood with increasing doses of the platelet agonists ADP, AYPGKF (PAR4) or CRP (GPVI) (Supplementary Figure S3). There were no indications of platelet pre-activation as activation markers of unstimulated platelets levels were <3% (Supplementary Figure S3).

### 3.2. PDE3A, but not PDE5, Inhibition Delays Platelet-Dependent Coagulation, while Maintaining Initial Haemostatic Thrombus Formation in Human and Mouse Platelets

Haemostatic platelet thrombus formation involves reciprocal interaction between platelets and coagulation factors [18]. To our knowledge, we are the first to assess the role of PDE3A and PDE5 on in vitro platelet thrombus formation under coagulating conditions using pharmacological inhibitors and blood from mice deficient in *Pde3a.* Notably, global *Pde5a* KO mice are embryonically lethal and no conditional *Pde5a* KO mouse models exist [19].

**Figure 2:**
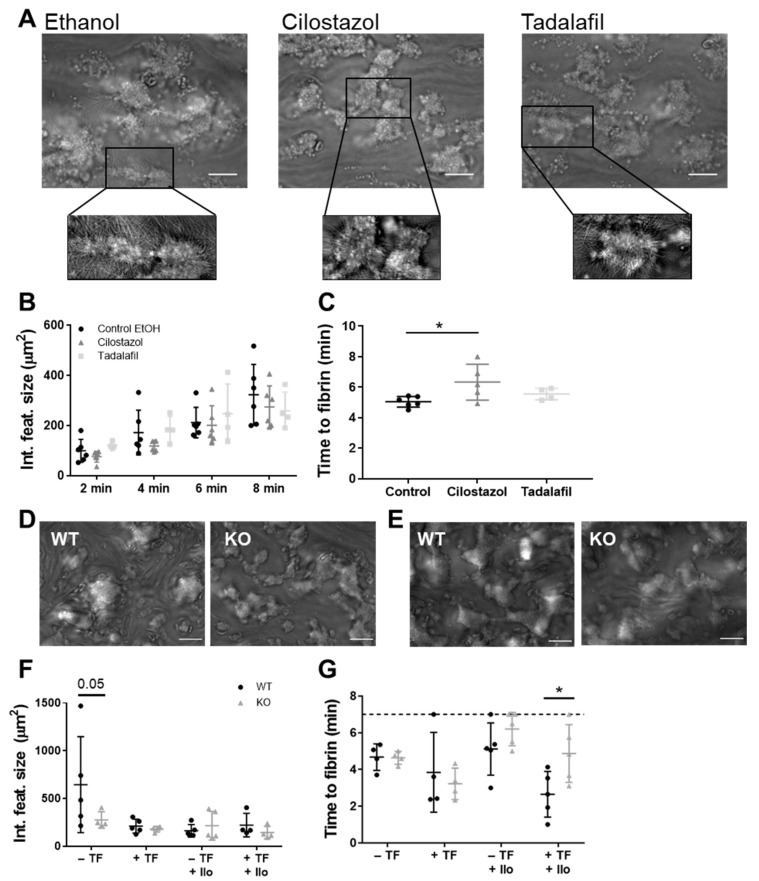
Effects of PDE inhibition or genetic deletion on platelet-dependent coagulation under flow over collagen. Recalcified citrate-anticoagulated human (**A**–**C**) or mouse (**D**–**G**) blood was perfused over a collagen type I surface or a combined collagen type I plus tissue factor surface for 7 (mouse) or 8 (human) min at a wall shear rate of 1000 s^−1^. (**A**) Representative brightfield images after 8 min of blood perfusion without inhibitor (0.1% ethanol) or in the presence of cilostazol (50 μM) or tadalafil (100 nM). Quantitative analysis of integrated feature size (μm) (**B**) and time to fibrin formation (**C**). Representative brightfield images of blood perfusion of WT mice and *Pde3a* KO mice over collagen type I without (**D**) or with (**E**) tissue factor in the absence of iloprost under coagulating conditions. Quantitative analysis of integrated feature size (**F**) and time to fibrin (**G**). Scale is 20 μm. Mean ± S.D., *n* = 4–6 (human) or 4–5 (mouse), * *p* < 0.05. Statistics: two-way ANOVA followed by Dunnett’s (**B**) or Sidak’s (**F**,**G**) multiple comparisons test. Time to fibrin formation (human) was tested by ordinary one-way ANOVA followed by Holm-Sidak’s multiple comparisons test (**C**). Ilo, iloprost; KO, knockout; TF, tissue factor; WT, wild type.

Upon perfusion, human platelets adhered instantaneously to the collagen I and tissue factor surface and formed large and fibrin-rich thrombi with an average time to fibrin formation of 302 s. Platelet-collagen interaction as such was not affected by cilostazol (50 μM, 95–98% protein-bound in plasma) or tadalafil (100 nM, 94% protein-bound in plasma) as the integrated feature size, which reflects platelet surface area coverage with respect to large thrombi and smaller platelet clusters [20], was unaltered (Figure 2A,B). Interestingly, cilostazol, but not tadalafil, significantly increased time to fibrin formation to 384 s (*p* = 0.03, Figure 2C). The surface area coverage of the platelet activation markers fibrin(ogen), CD62P and CD63 was unaltered (Supplementary Figure S4).

In a similar experimental setup, thrombus formation with blood from *Pde3a* KO mice was compared to that of WT mice (Figure 2D–F). Mouse thrombi were analysed based on their morphologic appearance, in which the integrated feature size was significantly decreased with blood *Pde3a* KO mice upon perfusion over the collagen type I surface, in the absence of tissue factor (*p* = 0.05, Figure 2F). When coagulation was stimulated by including the tissue factor in the collagen type I surface, the differences between WT and KO mice were abolished (Figure 2F). No phenotypic differences of thrombi were observed between genotypes (Supplementary Figure S5). To expand the experimental window for picking up an effect of PDE3 inhibition in mice, iloprost was added to the blood, which caused a raise in cAMP levels. In our hands, such an experimental window is smaller in mouse platelets when compared to human platelets as mouse platelets are more easily activated with, as a consequence, lower basal cAMP levels. Interestingly, the time to fibrin formation was increased in blood from *Pde3a* KO mice in comparison to blood from WT mice, in the presence of iloprost (Figure 2G). When investigating the activation markers of adherent platelets, the surface area coverage of JON/A, CD62P, or annexin V was found to be uniform among WT and *Pde3a* KO mice (Supplementary Figure S4). Altogether, these data suggest a promoting role for PDE3A, but not PDE5, in platelet-dependent fibrin formation, with platelet-collagen interaction under flow ranging from mildly inhibited to unaltered depending on the extent of coagulation.

### 3.3. PDE3A and PDE5 Inhibition Decreases Platelet Adhesion to Inflamed Endothelial Cells

**Figure 3:**
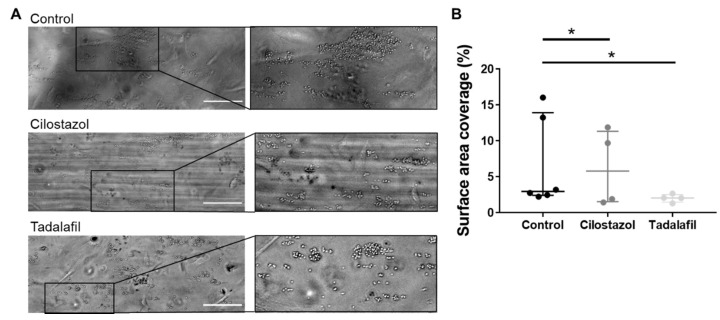
Platelet adhesion on inflamed endothelium is decreased upon PDE3A or -5 inhibition. Recalcified citrate-anticoagulated human blood was perfused over HUVECs treated with 10 ng/mL TNF-α for 10 min at a wall shear rate of 1000 s^−1^. (**A**) Representative brightfield images after 10 min of blood perfusion without inhibitor (0.1% ethanol) or in the presence of cilostazol (5 μM) or tadalafil (10 nM). (**B**) Quantitative analysis of platelet surface area coverage. Scale is 100 μm. Interquartile range, *n* = 4–6, * *p* < 0.05. Statistics: paired *t*-test.

Next, we investigated the role of PDE3A under thromboinflammatory conditions, first specifically in platelet-endothelial interactions, in which different receptors are involved than in platelet interactions with vascular matrix components [21,22]. Healthy endothelial cells ensure platelet quiescence, among others via the secretion of prostacyclin and nitric oxide [23], and indeed, hardly any platelet adhesion was visible during and after whole blood perfusion over untreated endothelial cells (Supplementary Figure S6). Inflammatory conditions were created by overnight or 4 h stimulation of HUVECs with TNF-α (10 ng/mL), which gave similar results (Supplementary Figure S6). Whole blood perfusion over inflamed endothelial cells resulted in the adhesion of single platelets, which clustered but remained a single layer of platelets (Figure 3A). Pre-treatment of blood from healthy volunteers with cilostazol reduced platelet adhesion on the inflamed endothelium, already at a dosage which is 10-fold lower (5 µM, Figure 3B) than was used for the platelet-collagen interaction experiments (Figure 2). Interestingly, incubation with 10 nM tadalafil, which is a 10-fold lower dose than the one used for platelet-collagen interaction under flow (Figure 2), inhibited platelet-endothelial interaction to a similar extent as cilostazol. These data suggest that pharmacological inhibition of PDE3A and PDE5 inhibits interactions of platelets with inflamed endothelium.

### 3.4. Monocyte Migration to and Adhesion on Platelets Is Reduced by Inhibition of PDE3A but not of PDE5

**Figure 4:**
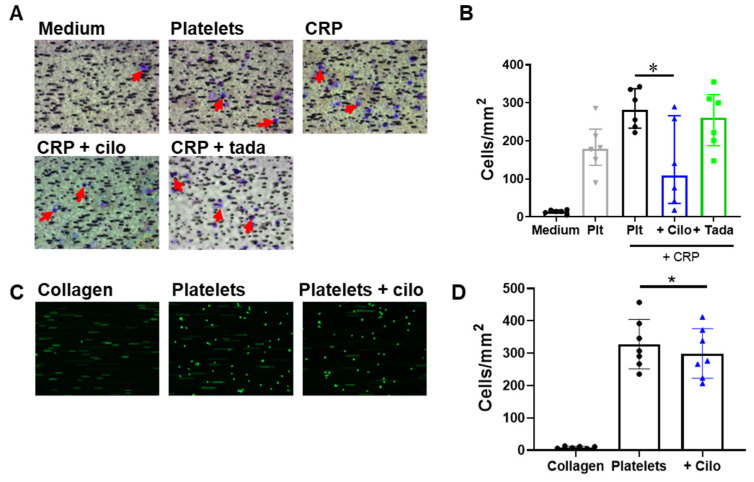
PDE3A inhibition decreases platelet-induced migration and platelet-induced adhesion of THP-1 cells under flow. Representative images (**A**) and quantitative analysis (**B**) of THP-1 cell migration to medium and to washed platelets, untreated or additionally stimulated with CRP and inhibited with cilostazol (5 μM) or tadalafil (10 nM). Red arrows indicate THP-1 cells. Representative images (**C**) or quantitative analysis (**D**) of THP-1 adhesion to collagen alone, and to washed platelets in the absence or presence of cilostazol (5 μM). Interquartile range (**B**), mean ± S.D. (**D**), *n* = 6 (**A**,**B**) or 7 (**C**,**D**), * *p* < 0.05. Statistics: Kruskal–Wallis test followed by Dunn’s multiple comparisons test (**B**) and Wilcoxon matched-pairs signed rank test (**D**). Cilo, cilostazol; CRP, collagen-related peptide; plt, platelets; tada, tadalafil.

In the context of (thrombo)inflammation, platelets facilitate monocyte activation and migration into the endothelium via chemotaxis and via direct interaction with monocytes [1,24]. Platelet-induced monocyte migration was studied with a chemotaxis chamber (Figure 4). Washed platelets already induced migration of THP-1 cells without the addition of a platelet agonist (Figure 4A,B), which is suggestive of platelet activation in the well. Platelet activation with CRP showed a positive trend (*p* = 0.11) to further increased THP-1 cell migration. Cilostazol (5 μM) pre-treatment diminished the CRP-induced chemotaxis, whereas PDE5 inhibition with tadalafil showed no effect (Figure 4B). Subsequently, we studied the effect of PDE3A inhibition on monocyte adhesion to a platelet monolayer, formed on a collagen type I surface, under flow conditions. Inhibition of PDE3A caused a relatively small, but significant (*p* = 0.04), reduction in THP-1 adhesion to platelets (Figure 4C,D). Taken together, inhibition of PDE3A and thus increasing cAMP in platelets decreased both platelet-induced monocyte migration as well as the adhesion of monocytes on platelets.

### 3.5. Platelet Chemokine- and Procoagulant EV Release Are Regulated by PDE3A and Partly by PDE5

**Figure 5:**
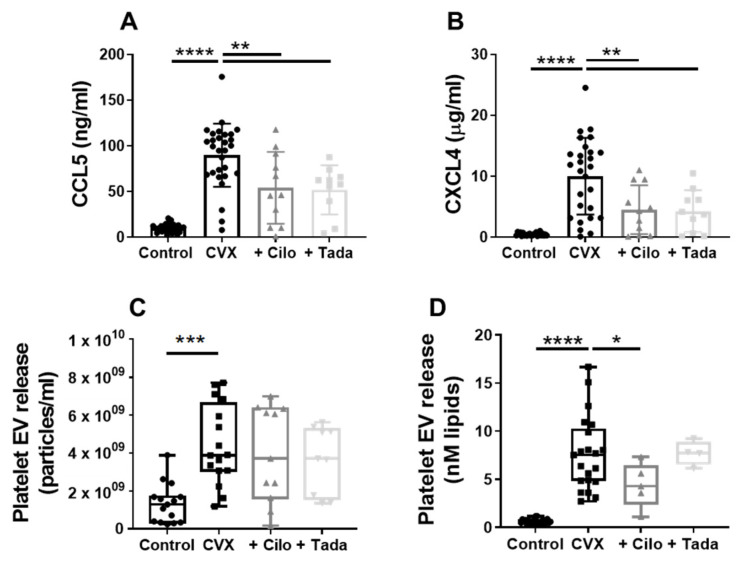
Effect of PDE3A and -5 inhibition on convulxin-induced chemokine and extracellular vesicle release by platelets. Washed platelets were stimulated with convulxin (100 ng/mL) without or with cilostazol (5 µM) or tadalafil (10 nM), and the release of chemokines CCL5 (**A**) and CXCL4 (**B**), and of total (**C**) and procoagulant (**D**) platelet extracellular vesicle (EV) was measured. (**A**,**B**) Mean ± S.D., *n* = 27–29 (control, CVX) or 10–11 (cilo, tada). (**C**,**D**) Interquartile range, *n* = 15–16 (control, CVX; NTA), 9–11 (cilo, tada; NTA), 21–22 (control, CVX; PTase), 4–5 (cilo, tada; PTase), * *p* < 0.05, ** *p* < 0.01, *** *p* < 0.001, **** *p* < 0.0001. Statistics: ordinary one-way ANOVA followed by Dunnett’s (**A**,**B**) or Holm-Sidak’s (**C**,**D**) multiple comparisons test. Cilo, cilostazol; CVX, convulxin; NTA, nanoparticle tracking analysis; PTase, prothrombinase; tada, tadalafil.

Platelet chemokines promote leukocyte recruitment to the inflamed endothelium [1,24]. We examined the effect of PDE3A and PDE5 inhibition on the release of the chemokines CCL5 (RANTES) and CXCL4 (platelet factor 4) by platelets. Convulxin- or thrombin-activated platelets secreted a substantial amount of CCL5 and CXCL4 (Figure 5A,B, *p* < 0.0001, Supplementary Figure S7). Both cilostazol and tadalafil reduced this chemokine release (Figure 5A,B, *p* < 0.01, Supplementary Figure S7), implicating a thus far unexplored role for PDE3A and -5 signalling in the secretion of proinflammatory factors from platelets.

Platelet-derived EVs play an important role in both haemostasis and inflammation and other cardiovascular diseases [25,26]. NTA was used to measure total platelet EV release. Stimulation of platelets with convulxin or thrombin resulted in a 3.3-fold and 2.4-fold increase in EV release, respectively (Supplementary Figure S7C,D, *p* < 0.01). Neither cilostazol (5 μM) nor tadalafil (10 nM) affected the total platelet EV release induced by convulxin (Figure 5C) or thrombin (Supplementary Figure S7). Cells can shed various EV subtypes, among which procoagulant EVs have gained considerable attention [27]. Procoagulant (PS-positive) platelet EV release, determined with a prothrombinase-based assay and expressed as the amount of lipids (nM), was increased after platelet stimulation with convulxin (12.2-fold, Figure 5D, *p* < 0.01, Supplementary Figure S7D) or with thrombin (5.9-fold, Supplementary Figure S7, *p* < 0.001). PDE5 inhibition was not associated with altered procoagulant EV release. Importantly, cilostazol significantly decreased procoagulant EV release induced by convulxin (Figure 5D, *p* = 0.02), whereas it showed a tendency of reduction in procoagulant EVs after platelet stimulation with thrombin (Supplementary Figure S7, *p* = 0.08).

Taken together, the above findings suggest that the pathways of proinflammatory platelet functions are regulated through the actions of PDE3A and PDE5, although potentially in different ways.

## 4. Discussion

Platelets are increasingly considered to not only have a main role in haemostasis and thrombosis, but also to be important in other conditions, such as vascular inflammation [28,29]. In so-called thromboinflammatory diseases, mechanisms of thrombosis and inflammation are intertwined, and can elicit and amplify one another. Here we report yet unrecognised roles for PDE3A and PDE5 in platelet procoagulant and proinflammatory responses. We found that PDE3A, but not PDE5, promotes platelet-dependent coagulation, through the delay of platelet-dependent fibrin formation and the reduction in procoagulant platelet EV release. With respect to proinflammatory responses, we observed that PDE3A and PDE5 promote platelet chemokine release and the interaction of platelets with inflamed endothelium. Platelet PDE3A also promotes monocyte recruitment and platelet-monocyte interaction.

Pharmacological inhibition of both PDE3A and -5 led to an unaltered platelet surface area coverage, platelet integrin activation, and granule secretion on collagen type I under flow. This might seem in contrast to previous studies that found reduced thrombus volume and platelet aggregation under shear flow [30,31,32,33,34,35,36]. However, these studies were performed in the absence of coagulation. The experimental setting in our study encompassed coagulating conditions, which is an important difference in methodology, since thrombus formation and coagulation are interconnected by the interaction of platelets with coagulation factors [18]. For example, thrombin generated on the platelet thrombi, being a potent agonist, might dampen the effects of cilostazol on integrin activation, secretion, and thrombus formation. Notably, cilostazol did reduce integrin activation and secretion of α- and dense granules of washed platelets under noncoagulating conditions, which is in agreement with others [37,38,39].

Our pharmacological studies were complemented by ex vivo perfusion using whole blood from *Pde3a* KO mice. *Pde3a* KO platelets displayed normal integrin activation and thrombus formation, but an increased time to fibrin formation under coagulating conditions. These results correspond to the pharmacological PDE3A inhibition using cilostazol in human platelets and suggest that this delay in fibrin formation can be attributed to direct actions on PDE3A enzyme activity and not by e.g., blockage of adenosine reuptake, as observed with dipyridamole. Yet, in contrast to treatment of human platelets with cilostazol, we found integrin α_IIb_β_3_ activation and CD62P expression in flow cytometry to be unaltered in *Pde3a* KO platelets after stimulation with various platelet agonists. This might reflect experimental differences in responses of the washed human platelets compared to those in diluted mouse whole blood. Alternatively, the findings raise the question on the role of PDE3A in activation of mouse platelets versus human platelets. One explanation for our results might be different amounts of the enzymes in platelets; human platelets have on average 1400 copies of PDE3A and 10,900 copies PDE5 per platelet [40], and mouse platelets contain 3400 copies of PDE3A and 50,382 of PDE5 [41]. Evidence on the role of PDE3A in murine platelet activation is scarce in the literature. One study reported that cAMP concentrations in resting *Pde3a* KO platelets was twice as high as in WT counterparts, which would suggest that these *Pde3a* KO platelets have a higher activation threshold [42].

Previous studies have largely focused on the role of cilostazol on the (non-inflamed) endothelium with respect to platelet-endothelium interaction, but such data are lacking for tadalafil. Cilostazol was found to directly act on the endothelium by inducing NO production [43], and by suppressing expression of endothelial P-selectin and intercellular adhesion molecule-1 (ICAM-1) [44], which will dampen platelet activation and platelet-endothelial interaction [21]. Our findings demonstrate that cilostazol and tadalafil reduce the adhesion of platelets in whole blood to activated endothelial cells under arterial shear conditions. As endothelial cells also contain PDE3A and PDE5 and both inhibitors are present during the perfusion experiments, a role for endothelial PDE3/PDE5 in the reduced platelet-endothelium interaction with cilostazol and tadalafil cannot be excluded a priori. However, this would involve an instantaneous alteration of expressed receptors for platelet adhesion in activated endothelial cells, which is rather unlikely. Our findings with cilostazol extend those by Fukuoka et al. [45], who found that cilostazol inhibits platelet-endothelial cell interaction in murine cerebral microvessels after transient bilateral common carotid artery occlusion using infusion of labelled platelets obtained from a donor mouse. Of note, it is unclear from the article whether these donor platelets were also treated with cilostazol or not. In addition, elevation of cAMP in platelets by cilostazol was shown to reduce initial platelet accumulation at sites of laser-induced endothelial injury in vivo [46]. Taken together, our data suggest that platelet PDE3A and PDE5 do not only regulate thrombotic platelet responses, but also platelet function under inflammatory conditions.

During previous years, the importance of platelets in vascular inflammation and inflammatory diseases has been recognised, in which not only platelet-endothelial interactions are involved, but also the interplay between platelets and leukocytes [47,48]. Migration and adhesion of monocytes towards and onto platelets was decreased upon PDE3A inhibition, although the inhibiting effects observed for monocyte adhesion were not as pronounced as for monocyte migration. Our findings extend earlier work in which a similar reduction by cilostazol treatment in binding of monocytes to collagen-activated platelets was observed, albeit under static conditions in a flow cytometer [37]. In order to investigate the effects of PDE inhibition on the release of inflammatory mediators, we focused on the chemokines CCL5 and CXCL4, which are abundantly expressed in human platelets. In addition, the release of these chemokines correlates strongly with that of other α-granule associated proteins after activation [49]. Importantly, we show that platelet PDE3A inhibition resulted in a strong reduction of the release of chemokines CCL5 and CXCL4, which is in agreement with the reduction in α-granule release by PDE3 inhibition. These chemokines promote the adhesion of monocytes to endothelial cells [50,51]. Interestingly, PDE5 inhibition decreased the release of CCL5 and CXCL4, while leaving monocyte migration unaffected. Beyond monocyte recruitment, CCL5 and in particular CXCL4 are also involved in neutrophil recruitment and activation, T cell differentiation, and injury responses of vascular smooth muscle cells [52,53,54,55]. Thus, the inhibition of CCL5 and CXCL4 release from platelets might be beneficial for the prevention of these pathological effects. In summary, cilostazol inhibits monocyte recruitment to, and adhesion on, platelets, presumably via the reduced release of CCL5 and CXCL4. This might also extend to other chemokines released from platelets with relevance for vascular inflammation and remodelling, e.g., CXCL14 and CXCL12 [56,57]. For future studies, it would be of interest to implement modern OMICs to investigate whether PDE inhibition leads to changes in platelet lipid composition [58] or RNA contents [59].

In our study, neither cilostazol nor tadalafil altered total platelet EV levels measured by nanoparticle tracking analysis. Conflicting reports exist about the effect of cilostazol on platelet EV release [60]. No role of PDE3A inhibition in platelet EV release was found when this was measured with an ELISA [61,62]. In contrast, cilostazol decreased total platelet EV levels determined with flow cytometry [36,39,63,64,65,66]. The lack of consistent effects of cilostazol on EV release is presumably related to the method of characterization, emphasising the need for standardisation [26].

Platelet extracellular vesicles exert procoagulant properties [67,68]. The increased procoagulant activity in stroke and myocardial infarct patients is characterised by phosphatidylserine- (PS) or tissue factor expressing cells and extracellular vesicles [69,70,71,72]. We are the first to distinguish between total EV levels and the number of procoagulant EVs in the context of phosphodiesterase inhibition. We found reduced procoagulant (PS-positive) extracellular vesicle release by cilostazol but not by tadalafil. Interestingly, acetylsalicylic acid had no effect on either total EV release in stroke patients nor proinflammatory or procoagulant EV release [72]. A remaining question is how and to what extent our procoagulant EV fraction differs from our total EV fraction and therefore, an important next step would be to fully characterise the platelet extracellular vesicles using fluorescent markers to obtain an advanced perspective of distinct EV subtypes, in healthy subjects and in various platelet-associated diseases.

Taken together, our findings demonstrate that the PDE3A inhibitor cilostazol not only delays platelet-dependent fibrin formation and platelet procoagulant EV release, but also dampens platelet-mediated inflammatory responses. It would be of particular interest to examine whether cilostazol treatment also affects platelet (procoagulant) EVs in patients and whether the EV level is associated with (reduced) thrombotic events.

## 5. Highlights

- We identified novel roles for platelet PDE3A and PDE5 in promoting procoagulant and proinflammatory platelet functions.
- PDE3A inhibition with cilostazol reduced procoagulant extracellular vesicle release.
- Release of the chemokines CCL5 and CXCL4 was decreased by either cilostazol or the PDE5 inhibitor tadalafil.
- Our findings add mechanistic insight substantiating a beneficial effect of cilostazol in thromboinflammatory diseases.

## Data Availability

Data are available upon reasonable request from the corresponding author. Reagents and detailed methods of all procedures are provided in the Data Supplement.

## Supplementary Materials

Extensive methods and Supplementary Figures are outlined in the article supplement at https://www.mdpi.com/article/10.3390/cells10081998/s1. Figure S1: Flow cytometry gating strategy; Figure S2: Effects of PDE inhibition on VASP phosphorylation; Figure S3: Platelets from *Pde3a* KO mice show normal α_IIb_β_3_ integrin activation and α-granule secretion; Figure S4: Effects of PDE inhibition or genetic deletion on platelet activation markers under flow over collagen under coagulating conditions; Figure S5: *Pde3a* KO mice show normal platelet thrombus formation depending on morphology, contraction, and multilayer; Figure S6: Influence of the duration of TNF-α treatment of endothelial cells on platelet surface area coverage. Figure S7: Influence of PDE3A and -5 inhibition on thrombin-induced chemokine and extracellular vesicle release by platelets. Table S1: Effects of PDE inhibition on platelet cAMP and cGMP levels.

## References

[1] Coenen D.M., Heinzmann A.C.A., Karel M.F.A., Cosemans J.M.E.M., Koenen R.R. (2021). The multifaceted contribution of platelets in the emergence and aftermath of acute cardiovascular events. Atherosclerosis.

[2] Weitz J.I., Angiolillo D.J., Geisler T., Heitmeier S. (2020). Dual pathway inhibition for vascular protection in patients with atherosclerotic disease: Rationale and review of the evidence. Thromb. Haemost..

[3] Benjamin E.J., Virani S.S., Callaway C.W., Chamberlain A.M., Chang A.R., Cheng S., Chiuve S.E., Cushman M., Delling F.N., Deo R. (2018). Heart disease and stroke statistics-2018 update: A report from the American Heart Association. Circulation.

[4] World Health Organization Cardiovascular Diseases (cvds). https://www.Who.Int/en/news-room/fact-sheets/detail/cardiovascular-diseases-.

[5] Gresele P., Momi S., Falcinelli E. (2011). Anti-platelet therapy: Phosphodiesterase inhibitors. Br. J. Clin. Pharmacol..

[6] Gerhard-Herman M.D., Gornik H.L., Barrett C., Barshes N.R., Corriere M.A., Drachman D.E., Fleisher L.A., Fowkes F.G.R., Hamburg N.M., Kinlay S. (2017). 2016 AHA/ACC guideline on the management of patients with lower extremity peripheral artery disease: A report of the American College of Cardiology/American Heart Association task force on clinical practice guidelines. J. Am. Coll. Cardiol..

[7] Noma K., Higashi Y. (2018). Cilostazol for treatment of cerebral infarction. Expert Opin. Pharm..

[8] Toyoda K., Uchiyama S., Yamaguchi T., Easton J.D., Kimura K., Hoshino H., Sakai N., Okada Y., Tanaka K., Origasa H. (2019). Dual antiplatelet therapy using cilostazol for secondary prevention in patients with high-risk ischaemic stroke in Japan: A multicentre, open-label, randomised controlled trial. Lancet Neurol..

[9] Tamai Y., Takami H., Nakahata R., Ono F., Munakata A. (1999). Comparison of the effects of acetylsalicylic acid, ticlopidine and cilostazol on primary hemostasis using a quantitative bleeding time test apparatus. Haemostasis.

[10] Wilhite D.B., Comerota A.J., Schmieder F.A., Throm R.C., Gaughan J.P., Rao A.K. (2003). Managing PAD with multiple platelet inhibitors: The effect of combination therapy on bleeding time. J. Vasc. Surg..

[11] Florescu C., Mustafa E.R., Târtea E.A., Florescu D.R., Albu V.C. (2019). Antiplatelet therapy in secondary ischemic stroke prevention—a short review. Rom. J. Morphol. Embryol..

[12] Erdman M.K., Leary M.C. (2017). Antiplatelet agents: Mechanisms and their role in stroke prevention. Primer on Cerebrovascular Diseases.

[13] Bath P.M., Woodhouse L.J., Appleton J.P., Beridze M., Christensen H., Dineen R.A., Duley L., England T.J., Flaherty K., Havard D. (2018). Antiplatelet therapy with aspirin, clopidogrel, and dipyridamole versus clopidogrel alone or aspirin and dipyridamole in patients with acute cerebral ischaemia (TARDIS): A randomised, open-label, phase 3 superiority trial. Lancet.

[14] Powers W.J., Rabinstein A.A., Ackerson T., Adeoye O.M., Bambakidis N.C., Becker K., Biller J., Brown M., Demaerschalk B.M., Hoh B. (2019). Guidelines for the early management of patients with acute ischemic stroke: 2019 update to the 2018 guidelines for the early management of acute ischemic stroke: A guideline for healthcare professionals from the American Heart Association/American Stroke Association. Stroke.

[15] Gresele P., Zoja C., Deckmyn H., Arnout J., Vermylen J., Verstraete M. (1983). Dipyridamole inhibits platelet aggregation in whole blood. Thromb. Haemost..

[16] Parikh V., Bhardwaj A., Nair A. (2019). Pharmacotherapy for pulmonary arterial hypertension. J. Thorac. Dis..

[17] Masciarelli S., Horner K., Liu C., Park S.H., Hinckley M., Hockman S., Nedachi T., Jin C., Conti M., Manganiello V. (2004). Cyclic nucleotide phosphodiesterase 3A-deficient mice as a model of female infertility. J. Clin. Investig..

[18] Swieringa F., Spronk H.M.H., Heemskerk J.W.M., van der Meijden P.E.J. (2018). Integrating platelet and coagulation activation in fibrin clot formation. Res. Pract. Thromb. Haemost..

[19] Zhang M., Takimoto E., Hsu S., Lee D.I., Nagayama T., Danner T., Koitabashi N., Barth A.S., Bedja D., Gabrielson K.L. (2010). Myocardial remodeling is controlled by myocyte-targeted gene regulation of phosphodiesterase type 5. J. Am. Coll. Cardiol..

[20] de Witt S.M., Swieringa F., Cavill R., Lamers M.M.E., van Kruchten R., Mastenbroek T., Baaten C., Coort S., Pugh N., Schulz A. (2014). Identification of platelet function defects by multi-parameter assessment of thrombus formation. Nat. Commun..

[21] Coenen D.M., Mastenbroek T.G., Cosemans J.M.E.M. (2017). Platelet interaction with activated endothelium: Mechanistic insights from microfluidics. Blood.

[22] Versteeg H.H., Heemskerk J.W., Levi M., Reitsma P.H. (2013). New fundamentals in hemostasis. Physiol. Rev..

[23] Smolenski A. (2012). Novel roles of cAMP/cGMP-dependent signaling in platelets. J. Thromb. Haemost..

[24] Rayes J., Bourne J.H., Brill A., Watson S.P. (2020). The dual role of platelet-innate immune cell interactions in thrombo-inflammation. Res. Pract. Thromb. Haemost..

[25] Vajen T., Mause S.F., Koenen R.R. (2015). Microvesicles from platelets: Novel drivers of vascular inflammation. Thromb. Haemost..

[26] Dickhout A., Koenen R.R. (2018). Extracellular vesicles as biomarkers in cardiovascular disease; chances and risks. Front. Cardiovasc. Med..

[27] Gasecka A., Nieuwland R., Budnik M., Dignat-George F., Eyileten C., Harrison P., Huczek Z., Kapłon-Cieślicka A., Lacroix R., Opolski G. (2020). Randomized controlled trial protocol to investigate the antiplatelet therapy effect on extracellular vesicles (AFFECT EV) in acute myocardial infarction. Platelets.

[28] Franco A.T., Corken A., Ware J. (2015). Platelets at the interface of thrombosis, inflammation, and cancer. Blood.

[29] Koenen R.R. (2016). The prowess of platelets in immunity and inflammation. Thromb. Haemost..

[30] Yagi H., Yamaguchi N., Shida Y., Hayakawa M., Matsumoto M., Sugimoto M., Wada H., Tsubaki K., Fujimura Y. (2012). Cilostazol down-regulates the height of mural platelet thrombi formed under a high-shear rate flow in the absence of ADAMTS13 activity. Eur. J. Pharmacol..

[31] Minami N., Suzuki Y., Yamamoto M., Kihira H., Imai E., Wada H., Kimura Y., Ikeda Y., Shiku H., Nishikawa M. (1997). Inhibition of shear stress-induced platelet aggregation by cilostazol, a specific inhibitor of cGMP-inhibited phosphodiesterase, in vitro and ex vivo. Life Sci..

[32] Nakamura T., Uchiyama S., Yamazaki M., Iwata M. (2007). Synergistic effect of cilostazol and dipyridamole mediated by adenosine on shear-induced platelet aggregation. Thromb. Res..

[33] Onoda K., Ohashi K., Hashimoto A., Okuda M., Shimono T., Nishikawa M., Shimpo H. (2008). Inhibition of platelet aggregation by combined therapy with aspirin and cilostazol after off-pump coronary artery bypass surgery. Ann. Thorac. Cardiovasc. Surg..

[34] Ryu K.H., Han H.Y., Lee S.Y., Jeon S.D., Im G.J., Lee B.Y., Kim K., Lim K.M., Chung J.H. (2009). Ginkgo biloba extract enhances antiplatelet and antithrombotic effects of cilostazol without prolongation of bleeding time. Thromb Res..

[35] Tanigawa T., Nishikawa M., Kitai T., Ueda Y., Okinaka T., Makino K., Ito M., Isaka N., Ikeda Y., Shiku H. (2000). Increased platelet aggregability in response to shear stress in acute myocardial infarction and its inhibition by combined therapy with aspirin and cilostazol after coronary intervention. Am. J. Cardiol..

[36] Yamazaki M., Uchiyama S., Xiong Y., Nakano T., Nakamura T., Iwata M. (2005). Effect of remnant-like particle on shear-induced platelet activation and its inhibition by antiplatelet agents. Thromb. Res..

[37] Ito H., Miyakoda G., Mori T. (2004). Cilostazol inhibits platelet-leukocyte interaction by suppression of platelet activation. Platelets.

[38] Lee S.Y., Kang M.J., Cha J.K. (2008). Cilostazol reduces PAC-1 expression on platelets in ischemic stroke. J. Clin. Neurol..

[39] Nomura S., Inami N., Iwasaka T., Liu Y. (2004). Platelet activation markers, microparticles and soluble adhesion molecules are elevated in patients with arteriosclerosis obliterans: Therapeutic effects by cilostazol and potentiation by dipyridamole. Platelets.

[40] Burkhart J.M., Vaudel M., Gambaryan S., Radau S., Walter U., Martens L., Geiger J., Sickmann A., Zahedi R.P. (2012). The first comprehensive and quantitative analysis of human platelet protein composition allows the comparative analysis of structural and functional pathways. Blood.

[41] Zeiler M., Moser M., Mann M. (2014). Copy number analysis of the murine platelet proteome spanning the complete abundance range. Mol. Cell Proteom..

[42] Sun B., Li H., Shakur Y., Hensley J., Hockman S., Kambayashi J., Manganiello V.C., Liu Y. (2007). Role of phosphodiesterase type 3A and 3B in regulating platelet and cardiac function using subtype-selective knockout mice. Cell. Signal..

[43] Hashimoto A., Miyakoda G., Hirose Y., Mori T. (2006). Activation of endothelial nitric oxide synthase by cilostazol via a cAMP/protein kinase A- and phosphatidylinositol 3-kinase/Akt-dependent mechanism. Atherosclerosis.

[44] Hase Y., Okamoto Y., Fujita Y., Kitamura A., Nakabayashi H., Ito H., Maki T., Washida K., Takahashi R., Ihara M. (2012). Cilostazol, a phosphodiesterase inhibitor, prevents no-reflow and hemorrhage in mice with focal cerebral ischemia. Exp. Neurol..

[45] Fukuoka T., Hayashi T., Hirayama M., Maruyama H., Tanahashi N. (2014). Cilostazol inhibits platelet-endothelial cell interaction in murine microvessels after transient bilateral common carotid artery occlusion. J. Stroke Cerebrovasc. Dis..

[46] Sim D.S., Merrill-Skoloff G., Furie B.C., Furie B., Flaumenhaft R. (2004). Initial accumulation of platelets during arterial thrombus formation in vivo is inhibited by elevation of basal cAMP levels. Blood.

[47] Projahn D., Koenen R.R. (2012). Platelets: Key players in vascular inflammation. J. Leukoc. Biol..

[48] van Gils J.M., Zwaginga J.J., Hordijk P.L. (2009). Molecular and functional interactions among monocytes, platelets, and endothelial cells and their relevance for cardiovascular diseases. J. Leukoc Biol..

[49] van Holten T.C., Bleijerveld O.B., Wijten P., de Groot P.G., Heck A.J.R., Barendrecht A.D., Merkx T.H., Scholten A., Roest M. (2014). Quantitative proteomics analysis reveals similar release profiles following specific PAR-1 or PAR-4 stimulation of platelets. Cardiovasc. Res..

[50] von Hundelshausen P., Koenen R.R., Sack M., Mause S.F., Adriaens W., Proudfoot A.E.I., Hackeng T.M., Weber C. (2005). Heterophilic interactions of platelet factor 4 and RANTES promote monocyte arrest on endothelium. Blood.

[51] Vajen T., Koenen R.R., Werner I., Staudt M., Projahn D., Curaj A., Sönmez T.T., Simsekyilmaz S., Schumacher D., Möllmann J. (2018). Blocking CCL5-CXCL4 heteromerization preserves heart function after myocardial infarction by attenuating leukocyte recruitment and NETosis. Sci. Rep..

[52] Rossaint J., Herter J.M., Van Aken H., Napirei M., Döring Y., Weber C., Soehnlein O., Zarbock A. (2014). Synchronized integrin engagement and chemokine activation is crucial in neutrophil extracellular trap-mediated sterile inflammation. Blood.

[53] Gerdes N., Zhu L., Ersoy M., Hermansson A., Hjemdahl P., Hu H., Hansson G.K., Li N. (2011). Platelets regulate CD4^+^ T-cell differentiation via multiple chemokines in humans. Thromb. Haemost..

[54] Shi G., Field D.J., Long X., Mickelsen D., Ko K.A., Ture S., Korshunov V.A., Miano J.M., Morrell C.N. (2013). Platelet factor 4 mediates vascular smooth muscle cell injury responses. Blood.

[55] Drechsler M., Megens R.T., van Zandvoort M., Weber C., Soehnlein O. (2010). Hyperlipidemia-triggered neutrophilia promotes early atherosclerosis. Circulation.

[56] Witte A., Chatterjee M., Lang F., Gawaz M. (2017). Platelets as a novel source of pro-inflammatory chemokine CXCL14. Cell Physiol. Biochem..

[57] Chatterjee M., von Ungern-Sternberg S.N.I., Seizer P., Schlegel F., Büttcher M., Sindhu N.A., Müller S., Mack A., Gawaz M. (2015). Platelet-derived CXCL12 regulates monocyte function, survival, differentiation into macrophages and foam cells through differential involvement of CXCR4-CXCR7. Cell Death Dis..

[58] Chatterjee M., Rath D., Schlotterbeck J., Rheinlaender J., Walker-Allgaier B., Alnagger N., Zdanyte M., Müller I., Borst O., Geisler T. (2017). Regulation of oxidized platelet lipidome: Implications for coronary artery disease. Eur. Heart J..

[59] Middleton E.A., Rowley J.W., Campbell R.A., Grissom C.K., Brown S.M., Beesley S.J., Schwertz H., Kosaka Y., Manne B.K., Krauel K. (2019). Sepsis alters the transcriptional and translational landscape of human and murine platelets. Blood.

[60] Rosinska J., Lukasik M., Kozubski W. (2017). The impact of vascular disease treatment on platelet-derived microvesicles. Cardiovasc. Drugs Ther..

[61] Ichijo M., Ishibashi S., Ohkubo T., Nomura S., Sanjo N., Yokota T., Mizusawa H. (2014). Elevated platelet microparticle levels after acute ischemic stroke with concurrent idiopathic thrombocytopenic purpura. J. Stroke Cerebrovasc. Dis..

[62] Shirafuji T., Hamaguchi H., Kanda F. (2008). Measurement of platelet-derived microparticle levels in the chronic phase of cerebral infarction using an enzyme-linked immunosorbent assay. Kobe J. Med. Sci..

[63] Chen Y., Xiao Y., Lin Z., Xiao X., He C., Bihl J.C., Zhao B., Ma X., Chen Y. (2015). The role of circulating platelets microparticles and platelet parameters in acute ischemic stroke patients. J. Stroke Cerebrovasc. Dis..

[64] Nomura S., Imamura A., Okuno M., Kamiyama Y., Fujimura Y., Ikeda Y., Fukuhara S. (2000). Platelet-derived microparticles in patients with arteriosclerosis obliterans. Thromb. Res..

[65] Nomura S., Shouzu A., Omoto S., Hayakawa T., Kagawa H., Nishikawa M., Inada M., Fujimura Y., Ikeda Y., Fukuhara S. (1998). Effect of cilostazol on soluble adhesion molecules and platelet-derived microparticles in patients with diabetes. Thromb. Haemost..

[66] Omoto S., Nomura S., Shouzu A., Hayakawa T., Shimizu H., Miyake Y., Yonemoto T., Nishikawa M., Fukuhara S., Inada M. (1999). Significance of platelet-derived microparticles and activated platelets in diabetic nephropathy. Nephron.

[67] Cauwenberghs S., Feijge M.A.H., Harper A.G.S., Sage S.O., Curvers J., Heemskerk J.W.M. (2006). Shedding of procoagulant microparticles from unstimulated platelets by integrin-mediated destabilization of actin cytoskeleton. FEBS Lett..

[68] Tans G., Rosing J., Thomassen M.C., Heeb M.J., Zwaal R.F., Griffin J.H. (1991). Comparison of anticoagulant and procoagulant activities of stimulated platelets and platelet-derived microparticles. Blood.

[69] Muller I., Klocke A., Alex M., Kotzsch M., Luther T., Morgenstern E., Zieseniss S., Zahler S., Preissner K., Engelmann B. (2003). Intravascular tissue factor initiates coagulation via circulating microvesicles and platelets. FASEB J..

[70] Sims P.J., Wiedmer T., Esmon C.T., Weiss H.J., Shattil S.J. (1989). Assembly of the platelet prothrombinase complex is linked to vesiculation of the platelet plasma membrane. Studies in Scott syndrome: An isolated defect in platelet procoagulant activity. J. Biol. Chem..

[71] Yao Z., Wang L., Wu X., Zhao L., Chi C., Guo L., Tong D., Yang X., Dong Z., Deng R. (2017). Enhanced procoagulant activity on blood cells after acute ischemic stroke. Transl. Stroke Res..

[72] Rosińska J., Maciejewska J., Narożny R., Kozubski W., Łukasik M. (2019). Association of platelet-derived microvesicles with high on-treatment platelet reactivity in convalescent ischemic stroke patients treated with acetylsalicylic acid. Wiad. Lek..

