# Supplementary material for: Inhibition of Phosphodiesterase 3A by Cilostazol Dampens Proinflammatory Platelet Functions
Source: Cells. 2021 Aug 5;10(8):1998. doi: 10.3390/cells10081998 (PMC8392606; doi:10.3390/cells10081998)
Supplement: Supplementary file 1 [file cells-10-01998-s001.zip › cells-1313941-supplementary.pdf]

**Supplementary methods**

***Reagents***

Cilostazol (Cas no. 73963-72-1) was ordered from Tebu Bio (Le-Perray-en-Yvelines, France) and tadalafil (Cas no. 171596-29-5) from Merck (Kenilworth, NJ, USA). Iloprost was bought in the hospital pharmacy of Maastricht University Medical Centre+ (MUMC+, Maastricht, The Netherlands) and sodium nitroprusside (SNP) from Janssen Pharmaceutica (Beerse, Belgium). Apyrase was obtained from Merck (Kenilworth, NJ, USA). Cross-linked collagen-related peptide (CRP) was purchased from CambCol Laboratories (Cambridge, UK), convulxin from Enzo (Zandhoven, Belgium), thrombin from Haematologic Technologies (Essex Junction, VT, USA) and collagen type I (HORM collagen) from Takeda (Tokio, Japan). 2-methylthio adenosine diphosphate (2-MeS-ADP) was bought from Santa Cruz Biotechnology (Dallas, TX, USA) and Ala-Tyr-Pro-Gly-Lys-Phe (AYPGKF) from Bachem Biosciences (Bubendorf, Switzerland). The monoclonal antibodies (mAbs) PAC-1, JON/A, anti-CD62P and anti-CD63 were purchased from Becton Dickinson (Franklin Lakes, NJ, USA), Emfret (Eibelstadt, Germany), Becton Dickinson (PE, Franklin Lakes, NJ, USA)/Beckman Coulter (FITC, Brea, CA, USA) and ITK Biolegend (San Diego, CA, USA), respectively. The mAbs against vasodilator-stimulated phosphoprotein (VASP) (phospho-Ser 157 and phospho-Ser 239) were purchased from Biomol (Hamburg, Germany). Fluorescently labelled fibrinogen and annexin V were purchased from Invitrogen Life Technologies (Thermofisher, Waltham, MA, USA). Tissue factor was ordered from Innovin (London, UK), D-Phe-Pro-Arg-chloromethylketone (PPACK) from Merck (Kenilworth, NJ, USA) and fragmin from Pfizer (New York, NY, USA). Calcium and magnesium were purchased from Merck (Kenilworth, NJ, USA) and Ambion (Thermofisher, Waltham, MA, USA). Saponin and formaldehyde were purchased from Merck (Kenilworth, NJ, USA). Human umbilical vein endothelial cells (HUVECs) were purchased from Lonza (Basel, Switzerland), THP-1 cells were from Leibniz-Institut DSMZ (ACC 16, Braunschweig, Germany), Endothelial Cell Growth Medium (ECGM) from Promocell (Heidelberg, Germany) and RPMI-1640 glutamax medium, fetal calf serum and penicillin/streptomycin were purchased from Gibco, Thermo Fisher (Waltham, MA, USA). Tumour necrosis factor-$\alpha$ (TNF-$\alpha$) was purchased from Merck (Kenilworth, NJ, USA).

***Platelet isolation***

Blood was drawn on 3.2% sodium citrate (Vacuette, Greiner bio-one). Before experiments or further sample processing, the haematological parameters were measured on a Sysmex XP-300 (Sysmex) to ensure that these were within normal range and that no platelet aggregates were present. Whole blood was centrifuged at 240 $g$ for 15 minutes without break to obtain platelet-rich plasma (PRP). Washed platelets were prepared by addition of 1:10 acid citrate dextrose (ACD, 80 mM trisodium citrate, 52 mM citric acid and 183 mM glucose) to the PRP and centrifugation at 2230 $g$ for 2 minutes. Subsequently, the platelet pellet was resuspended in Hepes buffer pH 6.6 (10 mM Hepes, 136 mM NaCl, 2.7 mM KCl, 2 mM MgCl2, 0.1% glucose and 0.1% bovine serum albumin (BSA)). After addition of 0.1 U/ml apyrase and ACD (1:15), the platelet suspension was centrifuged again at 2230 g for 2 minutes. The final platelet pellet was suspended in Hepes buffer pH 7.45. Platelet count was measured using a Sysmex XP-300. Presence of red and white blood cells and purity of washed platelets was verified by measurement of the haematological parameters on a Sysmex XP-300. Red blood cell concentration in the isolates ranged from 0.00–0.01 × $10^{12}$/L and the leukocyte concentration from 0.0–0.1 × $10^{9}$/L, with in a rare case 0.2 × $10^{9}$/L.

***Platelet activation with flow cytometry***

Human washed platelets (50 × $10^{6}$ platelets/ml) were incubated with a range of concentrations of cilostazol or tadalafil for 20 minutes. Next, the platelets were activated for 15 minutes with collagen-related peptide (CRP; 0.3 μg/ml) in the presence of 2 mM CaCl2 and $\alpha$IIbβ3 integrin activation and $\alpha$- and dense granule secretion were measured with PAC-1 (FITC, 1:20), anti-CD62P (FITC, 1:10) and anti-CD63 (APC, 1:20) antibodies with an Accuri C6 flow cytometer (Beckton Dickinson). An established and routine platelet gating strategy was used. SSC height was plotted against FSC-height. When

measuring isolated platelets, a dense population could be found, which was designated as the platelet gate (Supplementary Figure S 1 A). In washed platelet experiments, the run settings were set on measuring 5000 events, of which at least 99% were in the platelet gate. Next to that, the platelet population was inspected to confirm the purity of the washed platelet sample.

Mouse blood was diluted 25 times in Thyroid Hepes buffer pH 7.45 (5 mM Hepes, 136 mM NaCl, 2.7 mM KCl, 0.42 mM NaH2PO4, 2 mM MgCl2, 0.1% glucose and 0.1% BSA) with addition of 20 µM PPACK and 20 U/ml fragmin. Diluted blood was incubated for 4 minutes with 5 nM iloprost and activated for 10 minutes with a concentration range of AYPGKF (50 µM, 75 µM and 100 µM), CRP (0.5 µg/ml, 1 µg/ml and 10 µg/ml) or 2-MeS-ADP (0.01 µM and 0.1 µM) in the presence of 2 mM CaCl2. $\alpha$IIbβ3 integrin activation and $\alpha$-granule secretion were measured with JON/A (PE, 1:10) and anti-CD62P (FITC, 1:10) antibodies with an Accuri C6 flow cytometer. Here, the same platelet gate was used as with washed platelets. A control sample was run to optimize this gate and to exclude as many white and red blood cells as possible, of which the lower scatter is close to the larger scatter of the platelets. The run settings were then set on measuring 5000 events in this gate (Supplementary Figure S1B).

***cAMP/cGMP measurements***

Washed platelets (200 × $10^6$ platelets/ml) were incubated with cilostazol (5 µM) or tadalafil (10 nM) for 20 minutes or iloprost (10 nM) and sodium nitroprusside (SNP, 0.1 µM) for 2 minutes. Subsequently, the platelets were activated for 15 minutes with CRP (0.3 µg/ml) in the presence of 2 mM CaCl2. Ice-cold ethanol was added after which the samples were directly snap frozen in liquid nitrogen and stored at −80 °C until continuation of the assay. After thawing and centrifugation at 350 *g* for 10 minutes, the samples were dried under nitrogen at 56 °C and dissolved in assay buffer. cAMP and cGMP were measured using the Amersham Biotrak Enzymeimmunoassay (EIA) System (GE Healthcare Life Sciences) according to the manufacturer's instructions.

***VASP phosphorylation with flow cytometry***

Washed platelets (100 × $10^6$ platelets/ml) were incubated with cilostazol (5 µM) or tadalafil (10 nM) for 20 minutes or iloprost (10 nM) and SNP (0.1 µM) for 2 minutes. Subsequently, the platelets were activated for 15 minutes with CRP (0.3 µg/ml) in the presence of 2 mM CaCl2. Fixation was performed with 2% formaldehyde in phosphate-buffered saline (PBS) with 0.2% BSA for 15 minutes and the platelets were permeabilised with 0.1% saponin in PBS with 0.2% BSA after two centrifugation steps at 2230 *g* for 2 minutes and in between washing with PBS with 0.2% BSA. The fixated and permeabilised platelets were stained with anti-p-VASP S157 and S239 (FITC, 1:100) antibodies and analysed with an Accuri C6 flow cytometer (Beckton Dickinson).

***Collagen-mediated whole blood thrombus formation under flow***

GPVI-mediated thrombus formation under flow was performed using a parallel plate flow chamber (3000 × 50 µm) as described by de Witt et al. and Brouns et al [1,2]. Human blood was incubated with cilostazol (50 µM), tadalafil (100 nM) or vehicle (0.1% ethanol) for ten minutes. Recalcified blood (f.c. 6.3 mM CaCl2 and 3.2 mM MgCl2 (human) or 7.5 mM CaCl2 and 3.5 mM MgCl2 (mouse) was perfused for eight minutes at 1000 $s^{-1}$ over microspots that were coated with 50 µg/ml collagen type I and, where indicated, post-coated after one hour with 500 pM tissue factor. Because in our hands mouse platelets can more easily be activated than human platelets, the stable prostacyclin analogue iloprost (5 nM) was added as an extra condition in the murine perfusion experiments. Human blood was stained with fluorescently labelled fibrinogen (Alexa Fluor 546, 1:200) and with anti-CD62P (FITC, 1:40) and anti-CD63 (APC, 1:50) antibodies and murine blood with JON/A (PE, 1:20), anti-CD62P (FITC, 1:40) antibodies and annexin V (Alexa Fluor 647, 1:200). Brightfield and fluorescence images were taken during perfusion with an EVOS FL microscope with 60x magnification. Time to fibrin formation was determined by visual inspection of the formation of fibrin fibres from platelet thrombi. Analysis of the images was performed with Fiji ImageJ software. Images were scored based on morphology, contraction and multilayer.

***Platelet activation on and interaction with inflamed endothelium***

Platelet activation on and interaction with inflamed endothelium was examined using polydimethylsiloxane (PDMS) flow chambers (300 × 50 μm), made by standard soft lithography techniques. A mold was fabricated by patterning channel-like structures in SU-8 negative photoresist (Microchem) on a silicon wafer using photolithography. PDMS base and curing agent (Sylgard 184) were mixed at a 10/1 ratio (*w*/*w*) and degassed for two hours. The degassed PDMS was poured on the wafer and baked overnight at 60 °C. After the curing step the PDMS slab was removed from the wafer resulting in embossed channels in the PDMS slab. In- and outlets were made using a 1 mm biopsy puncher (Integra Miltex). Subsequently, the flow chambers were made by plasma treatment (Femto Science Cute, O2 plasma 50 W at 50 kHz and 40 seconds exposure) of the PDMS structures and a glass slide (Thermo Scientific) followed by bonding of the two layers. The channels were coated with a 100 μg/ml collagen type I (Corning, rat tail) solution in PBS for 30 minutes, after which HUVECs were seeded by introducing 10 μl cell suspension with a concentration of 15 × $10^6$ cells/ml in ECGM medium (PromoCell). After a 30-minute incubation, this step was repeated whilst incubating top-side down to coat both the bottom and the top of each channel. When confluency was reached, the cells were treated overnight or for 4 hours with TNF-$\alpha$ (10 ng/ml).

Recalcified blood (f.c. 6.32 mM CaCl2 and 3.16 mM MgCl2), incubated with cilostazol (5 μM), tadalafil (10 nM) or vehicle (0.1% ethanol) for ten minutes and stained with 1 μg/ml DiOC6, was perfused over the inflamed endothelial cell layer for ten minutes at 1000 $s^{-1}$. Subsequently, the channels were rinsed with Hepes buffer (supplemented with 200 mM CaCl2, 1 μg/ml heparin, 1% glucose and 1% BSA) and stained with anti-CD62P (PE, 1:40) and anti-CD63 (APC, 1:50) antibodies. Brightfield and fluorescence images were taken with an EVOS FL microscope with 20× magnification. Analysis of the images was performed with Fiji ImageJ software.

***THP-1 cell migration***

The principle of THP-1 cell migration was performed as described before [3]. In short, washed platelets (250 × $10^6$ platelets/ml) were incubated with cilostazol (5 μM) or tadalafil (10 nM) for 20 minutes, activated for 15 minutes with CRP (1.5 μg/ml) in the presence of 2 mM CaCl2 and applied to the bottom wells of a 12-well chemotaxis chamber (Neuro Probe). A polycarbonate membrane filter of 25 by 80 mm with 5 μm pores and a silicon gasket were carefully applied to prevent air bubbles and the top 12-well plate was placed. THP-1 cells (1 × $10^6$ cells/L) were added in the upper wells and the complete chamber was incubated at 37 °C with 5% CO2. After 90 minutes, the chamber was disassembled and non-migrated cells and debris on top of the filter were carefully removed. Subsequently, the filter was stained using a Diff-Quick staining (Eberhard Lehmann GmbH) and five representative images per well were made with a Leica DM2000 microscope with 40× objective. Analysis of the images was performed with Fiji ImageJ software.

***THP-1 cell adhesion***

Adhesion of THP-1 cells to a platelet monolayer was determined as previously described [4,5]. with minor adjustments. Briefly, a glass coverslip was coated with Horm collagen (50 μg/ml) for 1 hour at room temperature. Subsequently, the coverslip was blocked for 30 minutes with 1% BSA in Hepes buffer pH 7.45 and mounted into an Ibidi sticky-Slide VI 0.4. The channels were incubated with washed platelets (20 × $10^6$ platelets/ml, isolated as described above) for 1 hour at 37 °C. After removing non-adherent platelets and blocking the channel with 5% BSA in Hepes buffer pH 7.45 for 30 minutes at room temperature, platelets were inhibited with 5 μM cilostazol for 20 minutes at 37 °C. Perfusion of fluorescently labelled leukocytes was performed at a shear rate of 0.2 dynes/$cm^2$ (0.2 ml/min).

***Platelet extracellular vesicle (EV) release***

Platelets were isolated from whole blood as described above with minor modifications. To obtain PRP, 1:15 ACD was added to the blood which was centrifuged at 350 *g* for 15 minutes without break. ThePRP was supplemented with ACD (1:10) and centrifuged at 1200 *g* for 15 minutes. The platelet pellet was resuspended in Hepes buffer pH 6.6 (with 0.2% glucose and 0.5% BSA) and centrifuged again at 1200 *g*

for 15 minutes after addition of 1:10 ACD. The final platelet pellet was suspended in Hepes buffer pH 7.45 (with 0.2% glucose and 0.5% BSA).

Washed platelets (200 × $10^6$ platelets/ml) were incubated with cilostazol (5 μM), tadalafil (10 nM) or vehicle (0.1% ethanol) for ten minutes at 37 °C, and stimulated with convulxin (100 ng/ml) or thrombin (5 nM) for 30 minutes at 37 °C. After centrifugation at 2520 *g* for 5 minutes, the supernatant was passed through a 0.8 μm hydrophilic Minisart syringe filter (Sartorius) to a fresh tube and centrifuged at 20,000 *g* for 1 hour at 4 °C. The supernatant was stored for chemokine analysis and the EV pellet was resuspended in Hepes buffer pH 7.5, snap frozen in liquid nitrogen and stored at −80 °C for further examination.

***Chemokine analysis***

CCL5 (RANTES) and CXCL4 (platelet factor 4) concentrations in platelet supernatant were measured using an enzyme-linked immunosorbent assay (ELISA) kit from R&D systems according to the manufacturer's instructions. The biotinylated goat anti-human detection antibody for CCL5 was made in-house from sera of goats immunized with human CCL5 at Eurogentec sarl (Seraing, Belgium).

***Platelet EV analysis***

Platelet EV concentration was measured with nanoparticle tracking analysis (NTA) using the Nanosight NS300 (Malvern Panalytical). NTA determines concentration and size of particles based on their Brownian motion [6]. Samples were diluted 1:20 in Hepes buffer pH 7.45 without BSA and measured under static conditions with a 488 nm laser and a camera level of 13. Three videos of 60 seconds each were captured per sample. Hepes buffer pH 7.45 with 0.5% BSA was measured to correct for the dissolvent of the samples.

Additionally, pro-coagulant platelet EV release was analysed with a prothrombinase-based assay. In this assay, formation of thrombin on a lipid surface is measured as described [7]. Here, the membrane of the platelet EVs served as the lipid surface. Briefly, the prothrombinase complex consisted of purified bovine coagulation factors Xa (0.05 nM) and Va (1 nM) in Hepes buffer pH 7.7 (25 mM Hepes, 150 mM NaCl, 5 mM CaCl2 and 0.5% BSA). Indicated amounts of platelet phospholipids or EVs were incubated for 10 minutes at 37 °C. Human prothrombin (500 nM, Haematologic Technologies) was added and subsamples were added to cold buffer pH 7.9 (50 mM Tris, 20 mM EDTA and 175 mM NaCl). Subsequently, thrombin substrate P2238 (Pepscan) was added and subsamples were measured in a plate reader every 30 seconds for 15 minutes at wavelengths of 405 nm and 490 nm at 37 °C. The lipid concentration was calculated from changes in the absorbance, compared to the lipid calibration curve.

## Supplementary table and figures

**Supplementary Table S1.** Effects of PDE inhibition on platelet cAMP and cGMP levels.

| | **cAMP** | | **cGMP** | |
|---|---|---|---|---|
| | **−CRP** | **+CRP** | **−CRP** | **+CRP** |
| **Control** | 1 ± 0.22 | 0.77 ± 0.17 | 1 ± 0.15 | 1.06 ± 0.57 |
| **Cilostazol** | 1.15 ± 0.21 | 1.05 ± 0.25 | 1.23 ± 0.42 | 1.47 ± 0.78 |
| **Tadalafil** | 1.08 ± 0.24 | 1.06 ± 0.23 | 1.38 ± 0.47 | 1.16 ± 0.65 |
| **Iloprost** | 9.92 ± 1.60 ** | | 0.68 ± 0.35 | |
| **SNP** | 1.34 ± 0.37 | | 7.4 ± 2.72 | |

Fold change of cAMP and cGMP levels of washed platelets, untreated or additionally stimulated with the agonist CRP (0.3 µg/ml) or the PDE inhibitors cilostazol (5 µM), tadalafil (10 nM) or the endothelium-like secretory products iloprost (10 nM) or sodium nitroprusside (0.1 µM). Mean ± S.E.M., $n$ = 3–10, ** $p < 0.01$. Statistics: Kruskal-Wallis test followed by Dunn's multiple comparisons test. CRP, collagen-related peptide; SNP, sodium nitroprusside.

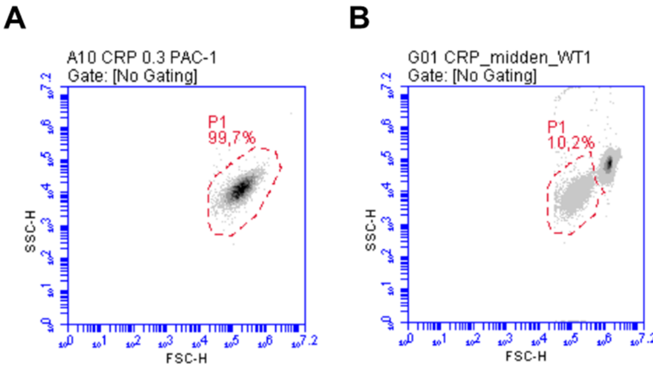


**Supplementary Figure S1.** Flow cytometry gating strategy. SSC height was plotted against FSC height. In a measurement with isolated platelets (**A**), a dense population could be found, designated as the platelet gate. In whole blood (**B**), the same gate was used and optimized to exclude as many white and red blood cells as possible, of which the lower scatter is close to the larger scatter of the platelets.

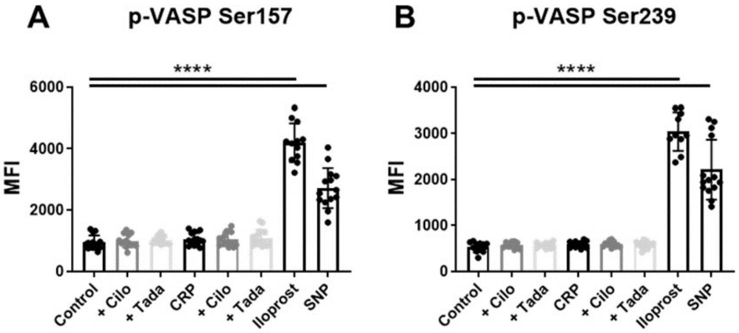


**Supplementary Figure S2.** Effects of PDE inhibition on VASP phosphorylation. Phosphorylation level, shown as median fluorescence intensity (MFI), of the VASP serine residue 157 (**A**) and the VASP serine residue 239 (**B**) is increased by the endothelium-like secretory products iloprost (10 nM) and sodium

nitroprusside (0.1 μM). Interquartile range, $n = 6$, **** $p < 0.0001$. Statistics: Kruskal-Wallis test followed by Dunn's multiple comparisons test. Cilo, cilostazol (5 μM); CRP, collagen-related peptide (0.3 μg/ml); SNP: sodium nitroprusside; tada, tadalafil (10 nM).

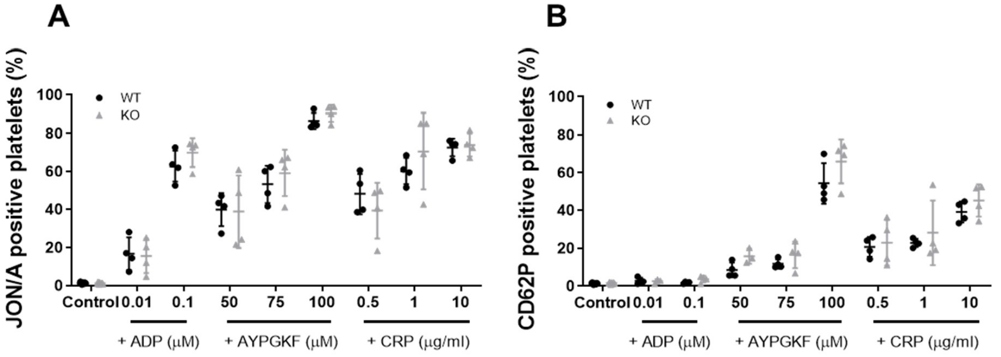


**Supplementary Figure S3.** Platelets from *Pde3a*$^{-/-}$ mice show normal αIIbβ3 integrin activation and α-granule secretion. Platelet integrin αIIbβ3 activation (**A**) and platelet α-granule secretion (**B**) were unaffected in *Pde3a*$^{-/-}$ mice compared with WT mice. Statistics: two-way ANOVA followed by Holm-Sidak's multiple comparisons test. Mean + S.D., $n = 4$. KO, knockout; WT, wild type.

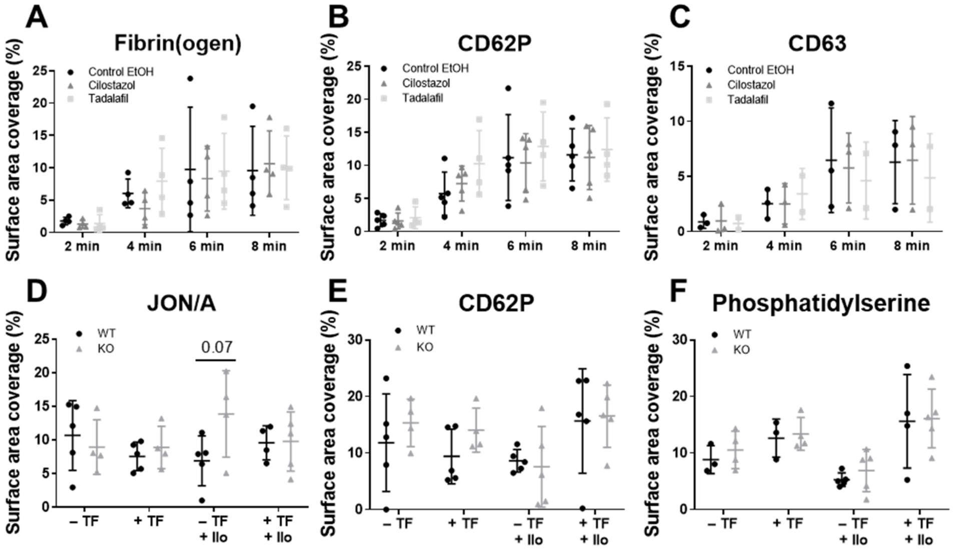


**Supplementary Figure S4.** Effects of PDE inhibition or genetic deletion on platelet activation markers under flow over collagen under coagulating conditions. Recalcified citrate-anticoagulated human (**A–C**) or mouse (**D–F**) blood was perfused over a collagen type I surface or a combined collagen type Iplus tissue factor surface for 7 (mouse) or 8 (human) min at a wall shear rate of 1000 $s^{-1}$. Human blood was perfused without inhibitor (0.1% ethanol) or in the presence of cilostazol (50 μM) or tadalafil (100nM). Quantitative analysis of surface area coverage of AF546-fibrin(ogen) (**A**), α-granule secretion (FITC-anti-CD62P) (**B**) and dense-granule secretion (APC-anti-CD63) (**C**) (human) and αIIbβ3 activation (PE-JON/A) (**D**), α-granule secretion (FITC-anti-CD62P) (**E**) and phosphatidylserine (PS) exposure (AF647-annexin A5) (**F**) (mouse). Mean ± S.D., $n = 2–5$ (human) or 4–5 (mouse). Statistics: two-way ANOVA followed by Dunnett's (**A–C**) or Sidak's (**D–F**) multiple comparisons test. Ilo, iloprost; KO, knockout; TF, tissue factor; WT, wild type.

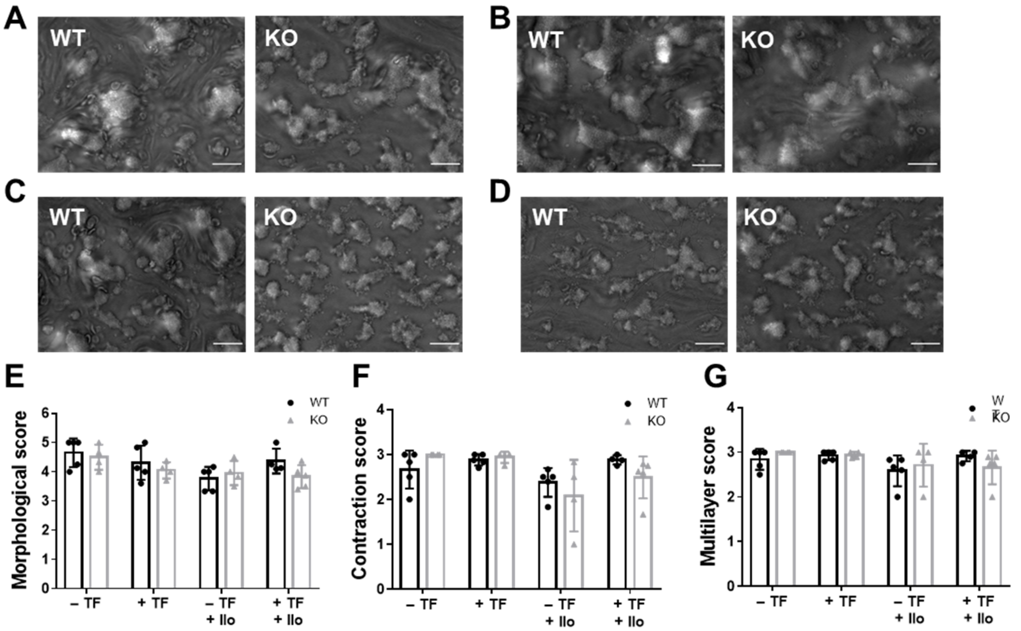


**Supplementary Figure S5.** *Pde3a*$^{-/-}$ mice show normal platelet thrombus formation depending on morphology, contraction, and multilayer. Recalcified citrate-anti-coagulated mouse blood was perfused over a collagen type I surface or a combined collagen type I plus tissue factor surface at a wall shear rate of 1000 s$^{-1}$. Representative images of blood perfusion of WT mice and *Pde3a*$^{-/-}$ mice over collagen type I without (**A**,**C**) or with (**B**,**D**) tissue factor under coagulating conditions in the absence (**A**,**B**) or presence (**C**,**D**) of iloprost (5 nM). Quantitative analysis of morphological score (from 0: no or hardly any adhered platelets to 5: large size thrombi) (**E**), contraction score (from 0: no contraction to 3: fully contracted) (**F**) and multilayer score (from 0: no multilayer to 3: large fully multilayered thrombi) (**G**). Scale is 20 µm. Mean ± S.D., $n$ = 4–5. Statistics: two-way ANOVA followed by Sidak's multiple comparisons test. Ilo, iloprost; KO, knockout; TF, tissue factor; WT, wild type.

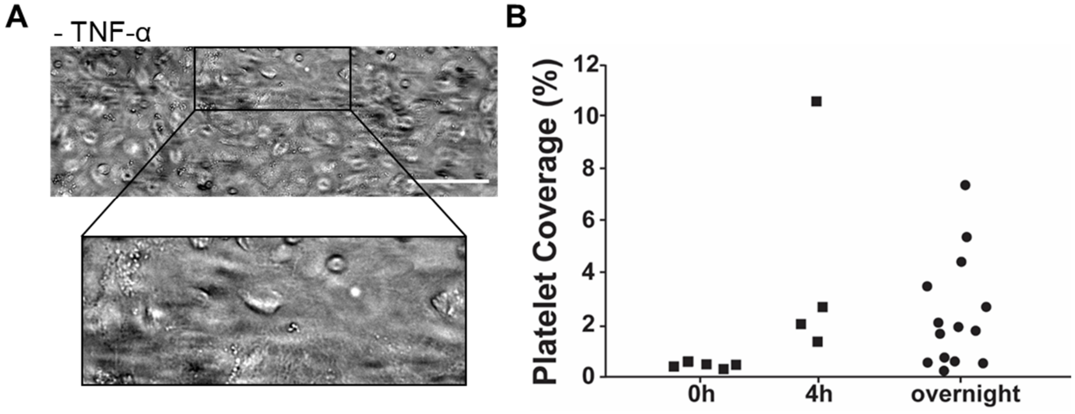


**Supplementary Figure S6.** Influence of duration of TNF-$\alpha$ treatment of endothelial cells on platelet surface area coverage. Recalcified citrate-anti-coagulated human blood was perfused for 10 minutes at a wall shear rate of 1000 s$^{-1}$ over human umbilical vein endothelial cells (HUVECs), untreated or stimulated with 10 ng/ml TNF-$\alpha$ either for 4 hours or overnight. (**A**) Representative brightfield image after 10 minutes of blood perfusion over untreated HUVECs. (**B**) Quantitative analyses of platelet surfacearea coverage. Scale is 100 µm. $n$ = 4–14.

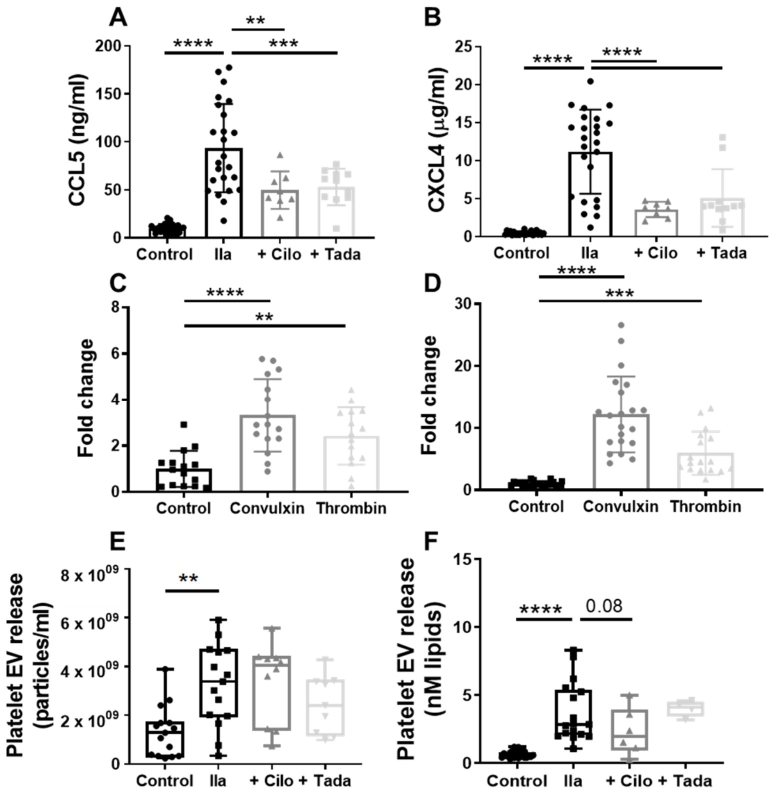


**Supplementary Figure S7.** Influence of PDE3A and -5 inhibition on thrombin-induced chemokine and extracellular vesicle release by platelets. Washed platelets were stimulated with thrombin (5 nM) without or with cilostazol (5 µM) or tadalafil (10 nM), and the release of chemokines CCL5 (**A**) and CXCL4 (**B**), and of total (**C,E**) and pro-coagulant (**D,F**) platelet extracellular vesicle (EV) was measured.(**A, B**) Mean ± S.D., $n$ = 24–27 (control, IIa) or 8-11 (cilo, tada). (**C,D**) Mean + S.D., $n$ = 15–16 (**C**), 17–22 (**D**). (**E,F**) Interquartile range, $n$ = 15 (control, IIa; NTA), 9-10 (cilo, tada; NTA), 17–22 (control, IIa; PTase), 4-6 (cilo, tada; PTase), ** $p < 0.01$, *** $p < 0.001$, **** $p < 0.0001$. Statistics: ordinary one-way ANOVA followed by Dunnett's (**A–D**) or Holm-Sidak's (**E,F**) multiple comparisons test. Cilo, cilostazol; IIa, thrombin; NTA, nanoparticle tracking analysis; PTase, prothrombinase; tada, tadalafil.

## Supplemental references


1. de Witt, S.M.; Swieringa, F. Cavill, R.; Lamers, M.M.E.; van Kruchten, R.; Mastenbroek, T.; Baaten, C.; Coort, S.; Pugh, N.; Schulz, A.; et al. Identification of platelet function defects by multi-parameter assessment of thrombus formation. *Nat. Commun*. **2014,** *5*, 4257.
2. Brouns, S.L.N.; van Geffen, J.P.; Campello, E.; Swieringa, F.; Spieza, L.; van Oerle, R.; Provenzale, I.; Verdoold, R.; Farndale, R.W.; Clemetson, K.J.; et al. Platelet-primed interactions of coagulation and anticoagulation pathways in flow-dependent thrombus formation. *Sci. Rep*. **2020**, *10*, 11910.
3. Vajen, T.; Benedikter, B.J.; Heinzmann, A.C.A.; Vasina, E.M.; Henskens, Y.; Parsons, M.; Maguire, P.B.; Stassen, F.R.; Heemskerk, J.W.M.; Schurgers, L.J.; et al. Platelet extracellular vesicles induce a pro-inflammatorysmooth muscle cell phenotype. *J. Extracell. Vesicles.* **2017**, *6*, 1322454.
4. Vajen, T.; Heinzmann, A.C.A.; Dickhout, A.; Zhao, Z.; Nagy, M.; Heemskerk, J.W.M.; Koenen, R.R. Laminar flow-based assays to investigate leukocyte recruitment on cultured vascular cells and adherent platelets. *J. Vis. Exp*. **2018**, 57009.
5. Zhao, Z.; Vajen, T.; Karshovska, E.; Dickhout, A.; Schmitt, M.M.; Megens, R.T.A.; von Hundelshausen, P.; Koeppel, T.A.; Hackeng, T.M.; Weber, C.; et al. Deletion of junctional adhesion molecule A from plateletsincreases early-stage neointima formation after wire injury in hyperlipidemic

mice. *J. Cell. Mol. Med.* **2017**, *21*, 1523-1531.
6. Dragovic, R.A.; Gardiner, C.; Brooks, A.S.; Tannetta, D.S.; Ferguson, D.J.P.; Hole, P.; Carr, B.; Redman, C.W.G.; Harris, A.L.; Dobson, P.J.; et al. Sizing and phenotyping of cellular vesicles using nanoparticle tracking analysis. *Nanomedicine* **2011**, *7*, 780-788.
7. Vasina, E.M.; Cauwenberghs, S.; Staudt, M.; Feijge, M.A.H.; Weber, C.; Koenen, R.R.; Heemskerk, J.W.M. Aging- and activation-induced platelet microparticles suppress apoptosis in monocytic cells and differentially signal to proinflammatory mediator release. *Am. J. Blood. Res.* **2013**, *3*, 107-123.
